# Prognostic relevance and putative histogenetic role of cytokeratin 7 and MUC5AC expression in Crohn’s disease-associated small bowel carcinoma

**DOI:** 10.1007/s00428-021-03109-2

**Published:** 2021-05-08

**Authors:** Giovanni Arpa, Alessandro Vanoli, Federica Grillo, Roberto Fiocca, Catherine Klersy, Daniela Furlan, Fausto Sessa, Sandro Ardizzone, Gianluca Sampietro, Maria Cristina Macciomei, Gabriella Nesi, Francesco Tonelli, Carlo Capella, Giovanni Latella, Antonio Ciardi, Roberto Caronna, Marco Vincenzo Lenti, Rachele Ciccocioppo, Valeria Barresi, Deborah Malvi, Antonietta D’Errico, Fernando Rizzello, Gilberto Poggioli, Claudia Mescoli, Massimo Rugge, Ombretta Luinetti, Marco Paulli, Antonio Di Sabatino, Enrico Solcia

**Affiliations:** 1 Unit of Anatomic Pathology, Department of Molecular Medicine, University of Pavia and Fondazione IRCCS San Matteo Hospital, Via Carlo Forlanini 16 –, 27100 Pavia, Italy; 2 Pathology Unit, Department of Surgical and Diagnostic Sciences, University Hospital and Ospedale Policlinico San Martino IRCCS, Genova, Italy; 3 Service of Clinical Epidemiology & Biometry, Fondazione IRCCS San Matteo Hospital, Pavia, Italy; 4 Anatomic Pathology Unit, Department of Medicine and Surgery, University of Insubria, Varese, Italy; 5 Gastroenterology, Luigi Sacco University Hospital, Milan, Italy; 6 ASST Rhodense, Rho Hospital, Rho, Italy; 7 Pathology Unit, San Camillo-Forlanini Hospital, Rome, Italy; 8 Division of Pathological Anatomy, Department of Surgery and Translational Medicine, University of Florence, Florence, Italy; 9 Department of Surgery and Translational Medicine, University of Florence, Florence, Italy; 10 Gastroenterology Unit, Department of Life and Environmental Sciences, University of L’Aquila, L’Aquila, Italy; 11 Department of Radiological, Oncological, Pathological Sciences, Umberto I Hospital, La Sapienza University, Rome, Italy; 12 Surgical Sciences, Umberto I Hospital, La Sapienza University, Rome, Italy; 13 Department of Internal Medicine, Fondazione IRCCS San Matteo Hospital, University of Pavia, Pavia, Italy; 14 Gastroenterology Unit, Department of Medicine, AOUI Policlinico G.B. Rossi, University of Verona, Verona, Italy; 15 Section of Anatomical Pathology, Department of Diagnostics and Public Health, University and Hospital Trust of Verona, Verona, Italy; 16 Department of Experimental, Diagnostic and Specialty Medicine (DIMES), Institute of Oncology and Transplant Pathology, St. Orsola-Malpighi Hospital, University of Bologna, Bologna, Italy; 17 Intestinal Chronic Bowel Disease Unit, Department of Medical and Surgical Sciences, Sant’Orsola Malpighi Hospital, Alma Mater Studiorum University of Bologna, Bologna, Italy; 18 Surgery of the Alimentary Tract, Department of Medical and Surgical Sciences, Sant’Orsola - Malpighi Hospital, Alma Mater Studiorum University of Bologna, Bologna, Italy; 19 Pathology Unit, Department of Medicine DIMED, University of Padua, Padova, Italy

**Keywords:** Small bowel adenocarcinoma, Cytokeratin 7, MUC5AC, Non-conventional dysplasia

## Abstract

Most Crohn’s disease-associated small bowel carcinomas (CrD-SBCs) are diagnosed in advanced stage and have poor prognosis. To improve diagnosis and therapy, a better knowledge of tumour precancerous lesions, histotypes and prognostic factors is needed. We investigated histologically and immunohistochemically 52 CrD-SBCs and 51 small bowel carcinomas unrelated to inflammatory disease, together with their tumour-associated mucosa, looking for Crohn-selective changes. Histologic patterns and phenotypic markers potentially predictive of CrD-SBC histogenesis and prognosis were analysed. Cytokeratin 7 or MUC5AC-positive metaplastic changes were found in about half of investigated CrD-SBCs, significantly more frequently than in CrD-unrelated SBCs. They correlated with metaplastic changes of their associated mucosa, while being absent in normal ileal mucosa. Histologic patterns suggestive for progression of some cytokeratin 7 and/or MUC5AC-positive metaplastic lesions into cancer of the same phenotype were also observed. Patient survival analyses showed that tumour cytokeratin 7 or MUC5AC expression and non-cohesive histotype were adverse prognostic factors at univariable analysis, while cytokeratin 7 and non-cohesive histotype were also found to predict worse survival in stage- and age-inclusive multivariable analyses. Besides conventional dysplasia, hyperplasia-like non-conventional lesions were observed in CrD-SBC-associated mucosa, with patterns suggestive for a histogenetic link with adjacent cancer. In conclusion the cytokeratin 7 and/or MUC5AC-positive metaplastic foci and the non-conventional growths may have a role in cancer histogenesis, while tumour cytokeratin 7 and non-cohesive histotype may also predict poor patient survival. Present findings are worth being considered in future prospective histogenetic and clinical studies.

**Supplementary Information:**

The online version contains supplementary material available at 10.1007/s00428-021-03109-2.

## Introduction

Most Crohn’s disease-associated small bowel carcinomas (CrD-SBCs) show unfavourable outcome due to a highly invasive pattern coupled with advanced stage at diagnosis [1–3]. Attempts to achieve an early diagnosis by the endoscopic screening of Crohn’s disease patients proved relatively ineffective compared with findings reported for early inflammatory bowel disease (IBD)-associated colorectal cancer [4–7]. This relative inability to endoscopically and histologically identify the precursors of CrD-SBC probably depends on the inability of endoscopy to routinely view the small intestine as well as on the limited knowledge of the histogenesis and natural history of CrD-SBCs. Indeed, there is some evidence that apparently non-dysplastic mucosa of cancer-bearing Crohn’s patients shows cancer-mimicking molecular changes [8, 9]. Therefore, efforts should be made to obtain a better characterization of putative cancer precursor lesions, alternative to or preceding established dysplasia, such as the long-known “pyloric” or “gastric” type metaplasia [10, 11], the “hyperplastic-like non-conventional type of dysplasia” [12] or the “ulcer-associated cell lineages (UACL)” [13, 14], previously reported in Crohn’s disease affected small bowel mucosa.

In a recent study on small bowel cancers, we were impressed by the large prevalence of non-intestinal tumour cell phenotypes among CrD-SBCs, partly mimicking gastric and/or pancreatobiliary duct cells, as well as by the occurrence, within their associated non-tumour mucosa, of focal changes showing the same “metaplastic” cell phenotypes as the cancer tissue itself [15]. We wondered whether such metaplastic changes may have a role in CrD-SBC histogenesis and progression, also considering previous suggestions concerning CrD-SBC [11, 16, 17] or IBD-promoted carcinogenesis [18]. In relation to this and in addition, we also considered the small bowel counterpart of the “non-conventional” low-grade dysplastic lesions, recently characterized among IBD patients with colorectal cancer [19].

In this study, we analysed the tumour cell phenotype, as well as putative precursor lesions in the background mucosa, of a large cohort of CrD-SBCs, collected and followed through the Small Bowel Cancer Italian Consortium and correlated these data with patients’ clinical outcome. Histologic and immunohistochemical findings in these cases were compared with those obtained in sporadically arising SBCs, in the absence of predisposing chronic immune-inflammatory diseases.

## Material and methods

### Study population

Fifty-two cases of CrD-SBCs (48 from the ileum, 2 from the jejunum and 2 from the duodenum), were collected and followed by 18 tertiary referral centres participating in the Small Bowel Cancer Italian Consortium. The patients had surgical resection with accurate tumour staging according to AJCC [20] and extensive tumour sampling; consequently, they entered clinical follow-up programs. In such CrD-SBC cases, the small bowel mucosa adjacent to the cancer (i.e., within 1 cm from cancer borders, in the same paraffin block) was also investigated to identify the presence of any metaplastic or non-conventional background lesion. In addition, 51 surgically resected SBCs (14 from the ileum, 31 from the jejunum and 6 from the duodenum) arisen sporadically in the absence of tumour predisposing immune-inflammatory disease such as coeliac disease or IBD (the no-PID-SBCs), together with their adjacent or distant non-neoplastic mucosa (beyond 1 cm from cancer), were histologically analysed as control group. The absence of any immuno-inflammatory disease in no-PID-SBC patients was ascertained by clinical findings, serology (for coeliac disease), CT abdominal scan and histologic investigation of non-tumour mucosa at distance from SBCs. All 8 duodenal cancers were non-ampullary. This study was approved by the Pavia Ethics Committee (protocol number 20140003980).

### Histology and immunohistochemistry

All cancers were classified histologically according to criteria previously found to be pathologically and clinically informative for SBCs [15, 21, 22]. The following main histotypes were considered: (a) glandular type, when 70% or more of the tumour exhibited a gland-forming pattern; (b) diffuse type, when 70% or more of the tumour showed diffusely infiltrating, poorly cohesive cells, with or without mucin-storing signet ring cells; or (c) mixed type, characterized by a combination of both glandular and diffuse/poorly cohesive patterns, each representing at least 30% of the tumour.

In accordance with previous studies [21–23], diffuse, mixed and a few solid-infiltrative cancers with cell clusters protruding from invasive edges, were classified as non-cohesive. Glandular cancers, solid cancers with no glands and luminal space, but retaining some cell polarity and basal membrane differentiation (trabecular pattern), and medullary carcinomas (characterized by expansive growth and abundant infiltration of T lymphocytes due to MSI status or Epstein-Barr virus aetiology [24]), were classified as cohesive. Tumour desmoplasia was considered only when the stroma was structurally rearranged, either with abundant newly formed cancer-associated fibroblasts (CAFs) or with consolidated fibrotic tissue, with or without myxoid or keloid patterns [23, 25].

For immunohistochemistry, 4-μm-thick tissue sections were stained using the following antibodies: CK7 (monoclonal, clone OV-TL 12/30, Dako), MUC5AC (monoclonal, clone CLH2, Abcam), MUC6 (monoclonal, clone CLH5, Novocastra), MUC2 (monoclonal, clone Ccp58, Santa Cruz Biotechnology), CDX2 (monoclonal, clone DAK-CDX2, Dako) and CK20 (monoclonal, clone Ks20.8, Dako); in order to assess tumour cell phenotype, for each one of the previously listed markers, only tumours with at least 10% of immunoreactive cells were regarded as positive, as previously reported [15, 26]. Cumulative intestinal marker expression was defined as expression of at least one marker among CDX2, MUC2 or CK20. In addition, protein p53 immunohistochemistry (monoclonal, clone DO7, Dako) was performed, and its expression was estimated as percentage of reactive cancer cells in SBCs and as scattered or widespread pattern in non-neoplastic tissues [2, 27].

For the assessment of mismatch repair (MMR)/MSI status, MLH1 (monoclonal, clone ES05, Dako), MSH2 (monoclonal, clone FE11, Dako), MSH6 (monoclonal, clone EP49, Dako) and PMS2 (monoclonal, clone EP51, Dako), immunostainings were performed and tumours were evaluated as MMR-proficient or MMR-deficient/MSI. Tumours were classified MMR-proficient when they retained the expression of MMR proteins and MMR-deficient/MSI (absent expression) when they lost MMR nuclear staining in the totality of neoplastic cells but not in the internal positive control (intra-tumour or peri-tumour inflammatory cells or non-neoplastic mucosa). Tumour-infiltrating lymphocytes (TILs) were stained using CD3 antibody (polyclonal, Dako, Carpinteria, CA) and quantified as previously described [3]. A tumour was considered as having “high TIL density” when the mean number of CD3+-positive TILs per high-power field was greater than 15.

Cancer-associated mucosae were evaluated for CK7 and MUC5AC expression, and they were scored as positive when at least one focus of more than five CK7-reactive or MUC5AC-reactive cells was found.

A central pathology review was performed by two gastrointestinal pathologists (AV and ES).

### Statistical analysis

The distribution of biomarkers was reported as counts and percentages, and Fisher’s exact test was used to compare them across clinical groups. Cumulative cancer-specific survival was plotted according to the Kaplan–Meier method. The cause of death was identified by the treating physician and/or from the corresponding code in the death certificate. The follow-up extended from the date of surgery to the date of death or last follow-up. The association of candidate prognostic factors and tumour-related death was estimated by means of univariable and multivariable Cox regression. Hazard ratios (HR) and 95% confidence intervals (CI) were computed. The following known non-collinear predictors of tumour death were included in the multivariable model in addition to CK7 or MUC5AC: stage, histotype and patient age at SBC diagnosis. Given the low number of events, no further adjustment for confounding factors could be performed. Hazard ratios and their 95% confidence intervals were computed. The proportional hazard assumption was tested, based on Schoenfeld residuals. A two-sided *P*-value <0.05 was considered statistically significant.

## Results

### Histology and phenotype of CrD-SBCs

**Fig. 1:**
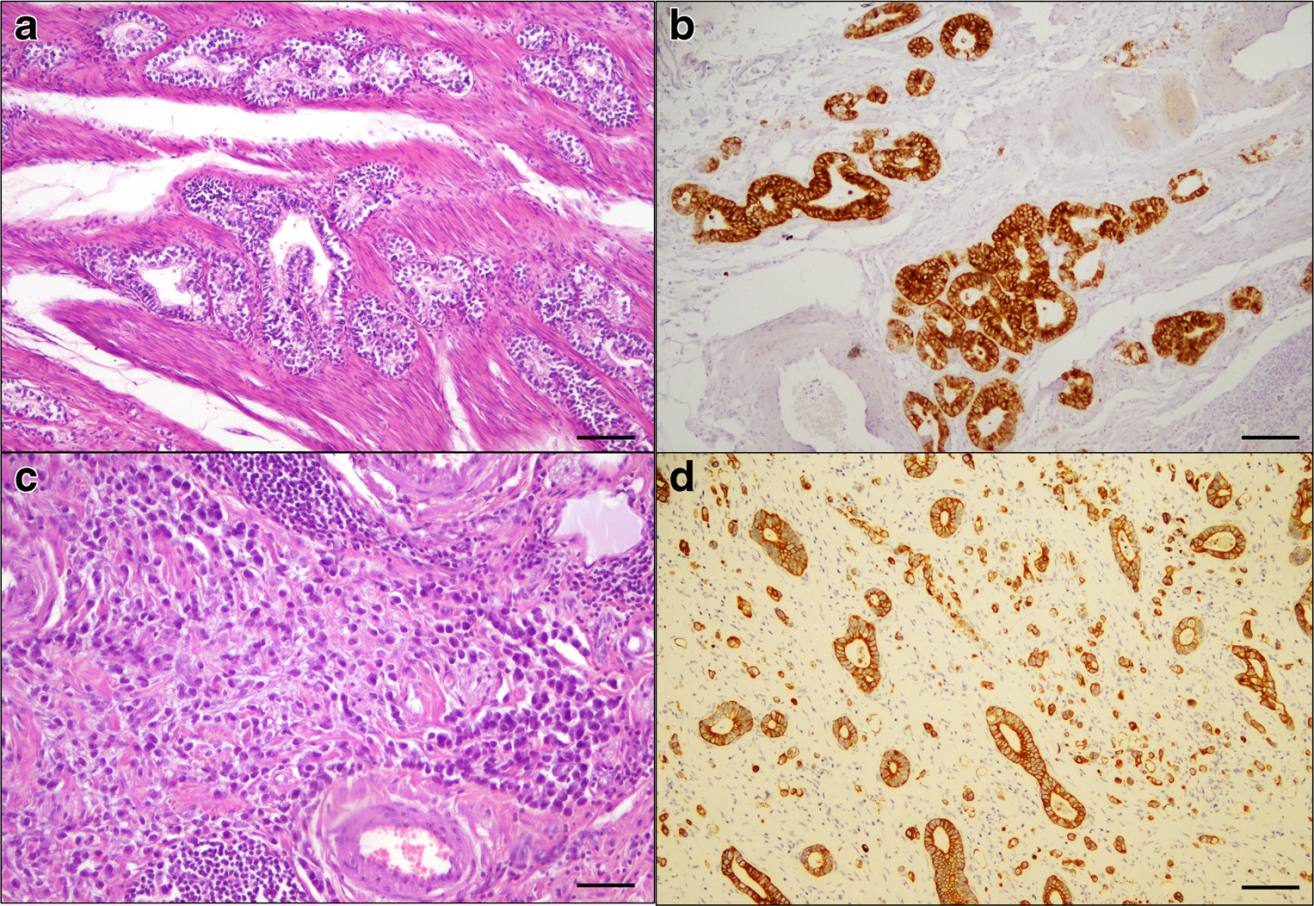
Histologic architecture and metaplastic phenotype of CrD-SBCs from the ileum. **a** A glandular type case invading the muscle fibres of the muscularis propria, which shows selective MUC5AC immunoreactivity in **b**. **c** A diffuse-type cancer is composed of small dispersed, undifferentiated cells, partly separated by minute desmoplasia. **d** A CK7-reactive mixed cancer shows an admixture of well-formed glands and dissociated cells or cell clusters infiltrating an abundant desmoplasia. **a** Haematoxylin and eosin staining, scale bar: 150μ; **b** MUC5AC immunohistochemistry, scale bar: 150μ; **c** haematoxylin-eosin staining, scale bar: 50μ; **d** CK7 immunoreactivity, scale bar: 150μ

CrD-SBCs were classified into *morphologic histotypes* (Fig. 1), as shown in Table 1. Twenty-nine cases were classified as cohesive, including 26 glandular type, one solid-trabecular and two solid-medullary cancers and 23 (44%) as non-cohesive, including 11 diffuse and 12 mixed cancers, one of which showing a predominant solid-infiltrative pattern.

**Table 1:** Histotype and phenotype analysis of 52 Crohn’s disease-associated small bowel carcinomas

Histotype	*N* (%)	CK7+	MUC5AC+	MUC6+	Intestinal markers	
					CDX2+	Cumulative+
Cohesive, *N* (%)	29/52 (56)	14/29 (48)	13/29 (45)	7/29 (24)	12/29 (56)	24/29 (83)
Non-cohesive, *N* (%)	23/52 (44)	13/23 (56)	10/23 (43)	2/23 (9)	12/23 (52)	17/23 (74)
Diffuse, *N* (%)	11/52 (21)	2/11 (18)	3/11 (27)	0/11 (0)	7/11 (64)	9/11 (82)
Mixed, *N* (%)	12/52 (23)	11/12 (92)*	7/12 (58)	2/12 (17)	5/12 (42)	8/12 (67)
Total, *N* (%)	52/52 (100)	27/52 (52)^§^	23/52 (44)	9/52 (17)°	29/52 (56)	41/52 (79)

All markers were scored as positive if ≥10% of tumour cells were stained. For cumulative intestinal markers, expression of at least one marker among CDX2, MUC2 or CK20 was considered

*Significant difference in CK7 expression versus diffuse (*p*<0.001) and cohesive (*p*= 0.009) histotype

§No significant association with MUC5AC (*p*=0.16) or MUC6 (*p*=0.141) expression

°MUC6 expression was significantly less frequent (*p*<0.001) than that of CK7 or MUC5AC, while being associated (*p*=0.003) with MUC5AC expression

Data on the expression of intestinal (CDX2, CK20 and MUC2) and non-intestinal markers (MUC5AC, MUC6 and CK7) in 52 CrD-SBCs are shown in Table 1 and Supplementary Table 1. It appears that both CK7 and MUC5AC *metaplastic markers* were largely expressed in cancers, being present in 52% and 44% of cases, respectively, while as many as 67% of tumours (35 cases) showed at least one of the two markers and 29% (15 cases) both markers. Of interest was the significantly lower expression by tumour cells (in 9/52 cases, 17%) of MUC6, a specific marker of pyloric and Brunner glands. The three “canonical” intestinal markers CDX2, CK20 and MUC2 were individually expressed by 56%, 54% and 54% of tumours, respectively, with 79% of all cases showing at least one of such markers. Although the expression of metaplastic or canonical markers inside the same tumour cell or tumour area was often mutually exclusive, some hybrid cells expressing both types of markers were also found. Only a limited, non-significant association was observed between the CK7 and MUC5AC expression by tumour cells (*p*=0.16, Fisher’s exact test), while a significant association was found between MUC5AC and MUC6 expression (*p*=0.003), with all but one MUC6-positive cancers also showing MUC5AC reactivity. In addition, an inverse association was noted between the expression of CDX2 and that of CK7 (*p*=0.029) or MUC5AC (*p*<0.001).

CK7 turned out to be more expressed among mixed-type cancers (92%), while being significantly less expressed among purely diffuse cancers (18%, *p*<0.001) and among cohesive cancers (48%, *p*=0.009). A peculiarity of CK7 expression was its concentration in budding cells and in poorly differentiated clusters at the tumour invasive front of glandular cancers, a pattern suggesting worse behaviour [20, 28, 29]. Interestingly, 11 of the 12 mixed-type cancers also showed prominent desmoplasia, especially in their non-glandular component, while only 4 of the 11 purely diffuse cancers had desmoplasia (*p*=0.005), which was however not seen in the three signet-ring cell SBCs. Eight of 29 cohesive cancers showed relevant desmoplasia, usually at the tumour-invasive front. As a whole, considering the 23 tumours with desmoplasia, 19 of them (83%) also showed CK7 expression of their epithelial component.

p53 protein immunoreactivity of more than 50% of cancer cells was found in 27 of the 52 (52%) cases (Suppl Table 1). No significant correlation was observed with tumour histotype (*p*=0.365). Tumour MSI/MMR-deficiency was detected in 10 (19%) CrD-SBCs, only 5 of which with high TIL density.

### Comparison between CrD-SBC and no-PID-SBCs

Mean patient age at tumour diagnosis of the 52 CrD-SBCs was 57.6 ±12.9 years, significantly lower than that of no-PID-SBC cases (69±13.4 years, *p*<0.001), in the absence, however, of significant tumour stage differences (*p*=0.573). Only two diffuse-type cancers occurred among no-PID-SBCs, versus 11 among CrD-SBCs (*p*=0.008), while the non-cohesive histology accounted for only 25% of no-PID-SBC cases (Table 2) (versus 44% among CrD-SBC, *p*=0.046). By comparing each other Table 1 and Table 2, it clearly appears that CrD-SBCs express more frequently CK7 (*p*=0.008) and MUC5AC (*p*=0.044) than no-PID-SBCs. Notably, such differences remain highly significant (*p*=0.004 for CK7 and *p*=0.009 for MUC5AC) when the analyses were restricted to jejunal-ileal tumours only (i.e. 50 CrD-SBCs vs 45 no-PID-SBCs). No relevant difference was noted among the two etiologically different tumour groups concerning cumulative intestinal marker expression (*p*=0.556); however, the percentage of cases with CDX2 expression turned out to be significantly lower (*p*=0.007) in CrD-SBCs. MUC6 was rarely expressed in both groups.

**Table 2:** Histotype and phenotype analysis of no-PID-associated small bowel carcinomas

Histotype	*N* (%)	CK7+	MUC5AC+	MUC6+	Intestinal markers	
					CDX2+	Cumulative+
Cohesive, *N* (%)	38/51 (75)	6/38 (16)	6/38 (16)	3/23 (13)	32/38 (84)	36/38 (95)
Non-cohesive, *N* (%)	13/51 (25)*	7/13 (54)	6/13 (46)	1/10 (10)	9/13 (69)	10/13 (77)
Diffuse, *N* (%)	2/51 (4)**	1/2 (50)	0/2 (0)	1/2 (50)	2/2 (100)	2/2 (100)
Mixed, *N* (%)	11/51 (21)	6/11 (54)	6/11 (54)	0/8 (0)	7/11 (64)	8/11 (73)
Total, *N* (%)	51/51 (100)	13/51 (25)^§^	12/51 (24)^§§^	4/33 (12)	41/51 (80)^#^	46/51 (90)^##^

All markers were scored as positive if ≥10% of tumour cells were stained. For cumulative intestinal markers, expression of at least one marker among CDX2, MUC2 or CK20 was considered. Only 33 no-PID-SBCs were tested for MUC6, due to depletion of sufficient representative tumour sections

*Significant difference in non-cohesive histotype prevalence versus Crohn’s disease associated-small bowel carcinomas (CrD-SBCs) (*p*=0.046)

**Significant difference in diffuse histotype versus CrD-SBCs (*p*=0.008)

§Significant difference in CK7 expression versus CrD-SBCs (*p*= 0.008)

§§Significant difference in MUC5AC expression versus CrD-SBCs (*p*=0.044)

^#^Significant difference in CDX2 expression versus CrD-SBCs (*p*=0.007)

^##^No significant difference in cumulative intestinal marker expression versus CrD-SBCs (*p*=0.556)

p53 immunoreactivity of more than 50% of cancer cells was observed in 14 (42%) of no-PID-SBC cases investigated, without significant difference between the two groups (*p*=0.371). Tumour MSI/MMR deficiency was detected in 20 (39%) no-PID-SBC cases, a significantly higher frequency (*p*=0.031) than that of CrD-SBCs. In keeping with this finding, a high TILs density was more frequently (*p*=0.023) observed in no-PID-SBCs (14 cases, 70%), rather than in CrD-SBCs. Furthermore, in no-PID-SBCs only, an inverse correlation (*p*=0.01) was found between widespread p53 expression and MSI status.

### CrD-SBC and no-PID-SBC-associated non-neoplastic mucosa

#### Metaplastic lesions

**Fig. 2:**
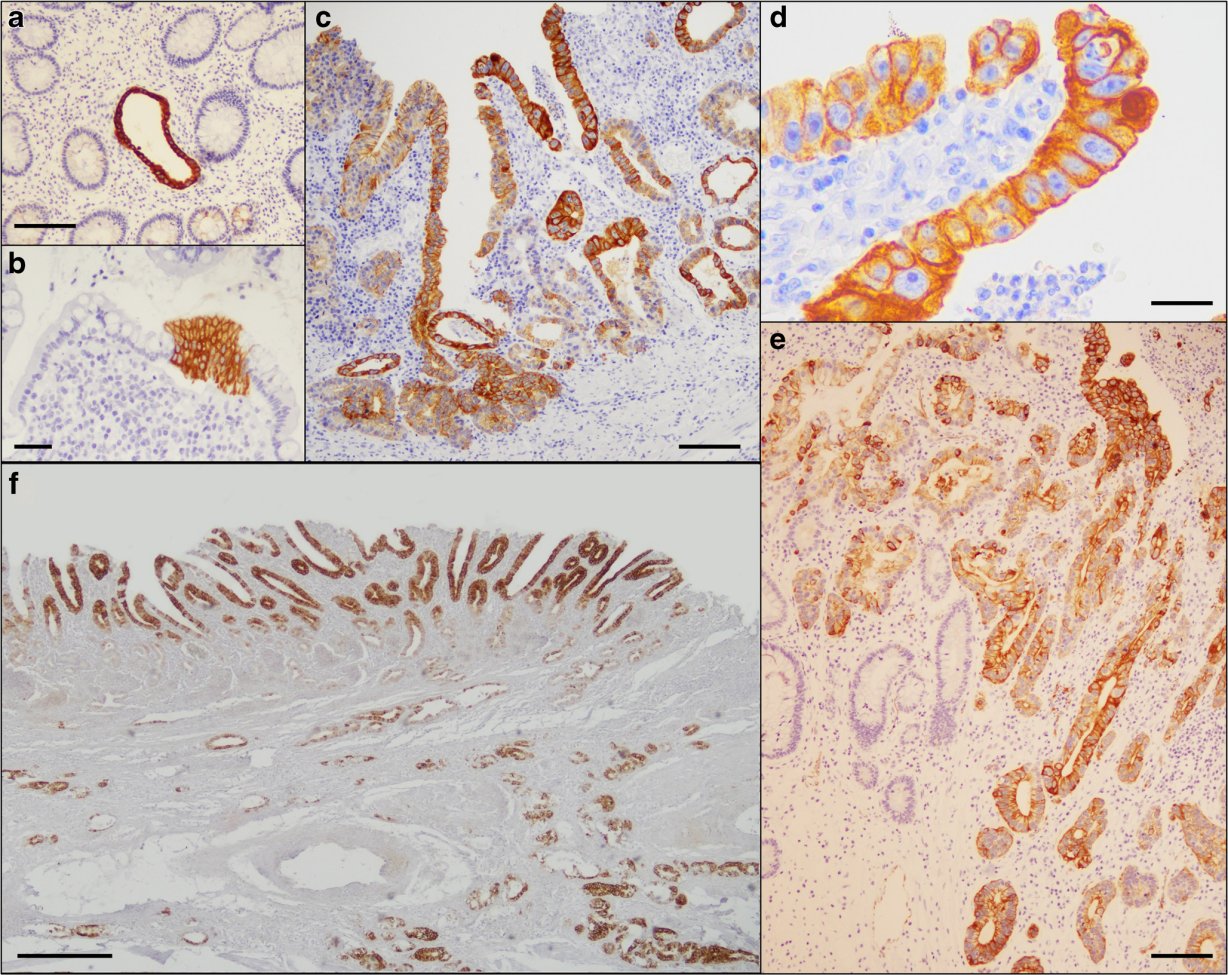
CrD-SBC-associated mucosa. **a** A single CK7-reactive ductular-like structure lacking topographic connection with, and differentiation signs towards, surrounding crypts. **b** A CK7-positive stratified button of unpolarized cells, lacking signs of cellular differentiation, abruptly interrupts the apparently normal epithelium of a villus. **c** CK7-reactive intra-epithelial cords and buttons of non-polarized, crowded cells with large nuclei and prominent nucleoli, up to frankly dysplastic foci (enlarged in **d**). **e** A diffusely CK7-reactive, perturbed metaplastic epithelium (in the upper left corner) with patterns suggestive for seamless transition to an atypical epithelial button (in the upper right corner) and to a glandular-type invasive cancer (in the bottom right). **f** An extensive superficial foveolar-type (MUC5AC-positive) lesion covers an underlying invasive cancer, also reactive, although less intensely, for MUC5AC. **a–e** Cytokeratin 7 immunohistochemistry, scale bars for **a**, **c**, **e**: 150μ; scale bar for **b**: 50μ; scale bar for **d**: 20μ; **f** MUC5AC immunohistochemistry, scale bar: 500μ

In addition to conventional dysplastic lesions with well-known histology [1–7], CrD-SBC-associated mucosa showed MUC6-reactive well-differentiated pyloric-type glands, usually detected in the deeper mucosa, MUC5AC-reactive foveolar-type foci and CK7-reactive epithelial foci, scattered at various mucosal levels, with preference for ulcer borders and related regenerative epithelia (Fig. 2). Both CK7- and MUC5AC-reactive epithelia were significantly more frequent in non-neoplastic mucosae of CrD-SBC cases, in comparison with those of no-PID-SBCs (70% versus 19%, *p*<0.001, for CK7, and 59% versus 21%, *p*<0.001 for MUC5AC). CK7 and MUC5AC were frequently expressed by the same mucosa (24 out of 46 cases, 52%) and often associated with focal loss of intestinal markers. Such metaplastic foci were more frequently observed in juxta-tumoural areas, where patterns suggestive for direct transition to neoplasia of the same phenotype, as well as more atypical CK7-positive foci, with buttons or cords of non-polarized and crowded cells with large nuclei and prominent nucleoli, were occasionally identified. In addition, CK7-positive ductular-type structures unrelated to adjacent intestinal crypts or villi and lacking signs of cellular differentiation were identified. On the other hand, MUC6-positive pyloric-type glands, which usually showed a well-differentiated mucin-rich pattern lacking atypical changes, were as a rule CK7-negative, and most of them were MUC5AC-negative.

A detailed analysis of CK7 distribution among the non-neoplastic mucosa associated with CrD-SBCs or non-PID-SBCs is outlined in Table 3. It emerged that (i) CK7 was more frequently expressed by CrD-SBC mucosae in comparison with no-PID-SBC mucosa (*p*<0.001) and (ii) a highly significant (*p*<0.001) association existed between CK7 expression by CrD-SBCs and their respective mucosae, while such an association was lacking among no-PID-SBC cases. No CK7-reactive foci were observed in histologically normal jejunal-ileal mucosa from tumour-free sections of no-PID patients.

**Table 3:** Comparison of cytokeratin 7 expression in Crohn’s disease-associated and non-immune-inflammatory bowel disease-associated small bowel carcinomas with their adjacent non-tumour mucosa

A. CrD-SBCs (*p*<0.001)				B. No-PID-SBCs (*p*=1)			
	Mucosa+	Mucosa−	Total		Mucosa+	Mucosa−	Total
Cancer +	24	2	26	Cancer +	1	8	9
Cancer −	8	12	20	Cancer −	7	27	34
Total	32	14	46	Total	8	35	43

A high correlation (*p*<0.001) was found in CrD patients between CK7 expression by cancer and by adjacent mucosa; a correlation not found in no-PID patients

#### Non-conventional background lesions

While searching for metaplastic changes in CrD-SBC juxta-tumour mucosa, we observed several non-conventional lesions, often forming papillary-to-polypoid protrusions, with either mucinous or columnar eosinophilic cell differentiation. Direct continuity of such non-conventional, hyperplasia-like and mildly atypical lesions with cancer, without interposition of conventional adenomatous dysplasia, was ascertained in seven of the 46 cases investigated for this purpose. All, but one, cancers showed glandular histology and all, but two, strong and widespread (>50% of cells) p53 reactivity, while the precursor component lacked frank, conventional (adenomatous) dysplasia and showed only scattered p53 reactivity (Supplementary Fig. 1). CK7 and/or MUC5AC reactivity was found in 3 out of such 6 glandular cases.

Interestingly, a diffuse-type, signet ring cell cancer (MUC2-positive and p53-negative) was covered by a MUC2-reactive “hypermucinous” non-conventional lesion (Supplementary Fig. 2) resembling those described by Andersen and coworkers [30] in the colon of ulcerative colitis patients (even though a serrated pattern was absent in our case).

No non-conventional type growths comparable to those of CrD-SBC cases were seen in no-PID-SBC-associated mucosa.

### CrD-SBC patient survival

**Fig. 3:**
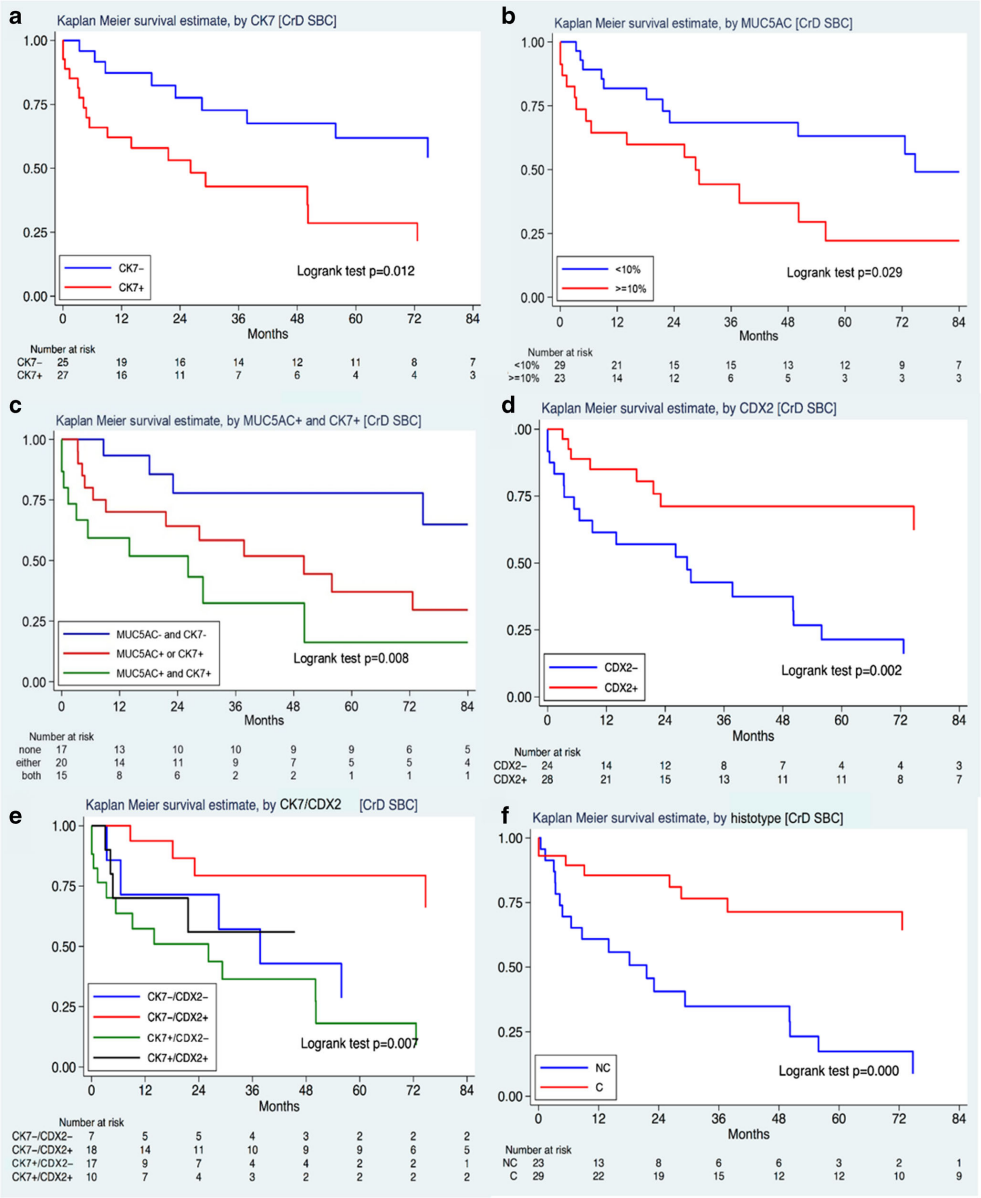
Kaplan-Meier survival estimates of CrD-SBC patients by CK7 (**a**), MUC5AC (**b**), combination of CK7 and MUC5AC (**c**), CDX2 (**d**), combination of CDX2 and CK7 (**e**) and cohesive versus non-cohesive histotype (**f**). CrD-SBC, Crohn’s disease-associated small bowel carcinoma; CK7, cytokeratin 7; C, cohesive; NC, non-cohesive

CrD-SBC patients were followed up for a median of 84.9 months (25th–75th: 31–121 months). Patients with CrD-SBC positive for CK7 had significantly worse cancer-specific survival compared to those with CrD-SBC negative for CK7 (HR: 2.72, 95% CI: 1.20–6.17, *p*=0.016; Fig. 3a), which was confirmed at stage-inclusive bivariable analysis (HR: 2.78, 95% CI: 1.21–6.38, *p*=0.016). At univariable analysis, MUC5AC-reactive cases also showed an association with a worse prognosis (HR 2.28, 95% CI: 1.04–5.04, *p*=0.038) (Fig. 3b). In addition, CrD-SBC patients with co-expression of MUC5AC and CK7 by the cancer (15 cases) featured a worse prognosis in comparison with those negative for both markers (17 cases) or with expression of either MUC5AC or CK7 (20 cases) (Fig. 3c).

The expression of at least one of the intestinal markers (CDX2, MUC2 or CK20) showed no significant correlation with patient survival (*p*=0.35). However, CDX2 expression alone was found to correlate with better overall survival (HR: 0.30, 95% CI: 0.13–0.68, *p*=0.004; Fig. 3d), although it lost prognostic power at stage-inclusive bivariable analysis (*p*=0.053). When CK7 and CDX2 expressions were combined, patients with CK7-positive and CDX2-negative tumours showed the worst outcome, while patients with CK7-negative and CDX2-positive tumours showed the best survival, with intermediate figures for the double-positive or double-negative tumours (Fig. 3e). None of the remaining markers tested, including MUC6 and p53, was significantly associated with patient survival.

An association of tumour non-cohesive histotype with worse patient survival was also observed (HR: 5.0, 95% CI: 1.82–10, *p*=0.001; Fig. 3f) and confirmed at stage-inclusive bivariable analysis (*p*=0.009), whereas MMR-d was not associated with patient cancer-specific survival (HR: 0.65, 95% CI: 0.22–1.92; *p*=0.434).

Importantly, a multivariable analysis including tumour CK7 (positive versus negative), histotype (cohesive versus non-cohesive), stage (I+II versus III+IV) and patient age at diagnosis showed an independent prognostic power for each of the four parameters (Table 4). MUC5AC, when included in the same model in place of CK7, lost predictive power of cancer-specific survival (HR 1.20, 95% CI: 0.49–2.91, *p*=0.692).

**Table 4:** Multivariable survival analysis of the 52 Crohn’s disease-associated small bowel carcinoma cases

Variable	Multivariable analysis*	
	HR (95% CI)	*P*-value
CK7+ (vs CK7−)	2.39 (1.02–5.58)	0.044
Cohesive histotype (vs non-cohesive)	0.20 (0.07–0.56)	0.002
Stage III/IV (vs I/II)	3.90 (1.53–9.92)	0.004
Age at cancer diagnosis (continuous)	1.05 (1.01–1.09)	0.019

*Multivariable model: *p*<0.001; Harrell’s *C*=0.8377

*CK7* cytokeratin 7, *CI* confidence interval, *HR* hazard ratio

## Discussion

In this study, we found that two non-intestinal markers, i.e. CK7 and MUC5AC, were expressed at significantly higher percentages in both tumour tissue and associated mucosa of CrD-SBC cases compared to ordinary no-PID-SBC cases. This finding, in addition to a significant increase among CrD-SBCs of the non-cohesive histotype and a lack of CK7 and MUC5AC expression in histologically normal ileal mucosa, suggests that a distinctive carcinogenic process plays a role in a relevant fraction of CrD-SBC cases. This hypothesis is reinforced by the highly significant association of CK7 expression between tumour tissue and respective mucosa, an association lacking in no-PID-SBC cases, as well as by the occasional finding of direct topographic continuity between CK7 and/or MUC5AC-positive mucosal lesions and the invasive cancers of the same phenotype, which we identified in some CrD-SBC cases. The additional observation that tumour CK7 positivity was an independent adverse prognostic factor points to some mechanism underlying the metaplastic growth which may favour both tumour development and progression.

Metaplastic changes of gastric type, with special reference to MUC6-positive “pyloric” glands, have long been reported in Crohn’s disease intestinal mucosa [10, 11]. While confirming this finding in non-neoplastic mucosa of CrD-SBCs cases, we found limited expression of MUC6 in corresponding cancer tissue and a lack of MUC6 prognostic influence, two observations rendering unlikely a significant contribution of MUC6-positive pyloric-type differentiation to CrD-SBC natural history. On the other hand, the involvement of the gastric foveolar cell marker MUC5AC is suggested by its high expression in both CrD-SBCs and nearby mucosa and by its association with worse patient outcome, in keeping with previous findings by others in SBC patients as a whole [31] or in the ampullary [32] and non-ampullary duodenal cancer patients [33].

CrD-SBC patient survival analysis highlighted an important prognostic influence of CK7-positive metaplastic phenotype. In particular, we found a significant association between CK7 expression by SBC and worse patient survival, which was confirmed at multivariable analysis inclusive of patient age, stage and histotype. Present findings suggesting a relationship between CK7 expression and CrD-SBC progression fit with previous observations on SBC as a whole [26] and on colorectal cancers [34, 35], where poor tumour differentiation, high tumour budding and increased invasive and metastatic potential were frequently observed in CK7-expressing cancers. No information is presently available on possible mechanisms involved in such tumour behaviour. Of interest is our finding of prominent desmoplasia associated with CK7-positive (especially mixed type) invasive cancers, as cancer desmoplasia and/or “mesenchymal” molecular subtype have been linked to worse prognosis in several digestive cancers, including gastric [36], pancreatic [37] and intestinal cancers [23, 25, 38–40]. Based on our findings, the MSI hyper-immune subtype, of known better prognostic value among various gastrointestinal cancers, including SBCs, [22–24] appears to be less represented and without prognostic relevance among CrD-SBCs.

In both CrD-SBCs and associated mucosa, metaplastic marker expression was commonly associated with loss of canonical intestinal markers, with special reference to the CDX2 transcription factor. This seems interesting as, in keeping with previous findings on gastrointestinal cancers, including SBCs as a whole [17, 41, 42], we found that CDX2 predicts improved patient survival. Although in the present CrD-SBC series it failed to remain significant at multivariable analysis due to collinearity with stage and histotype, this survival influence of CDX2 expression should be considered in light of the crucial role played by CDX2 in intestinal epithelium differentiation and metaplastic lineage development. Indeed, it has been recently shown in vitro by Simmini and coworkers [43] that loss of CDX2 expression alone is enough to transform intestinal stem cells into gastric lineages. Thus, the foci of CDX2 expression loss we found in ileal mucosa of Crohn’s patients might well be a starting point for the metaplastic changes occurring in vivo in this disease, either of gastric type (of pyloric, foveolar or their common precursor lineage) or of non-gastric CK7-expressing type. In this respect, the role of CK7 as a marker of gut foetal, embryonic or pluripotent stem cells [44–46] should also be considered, in addition to its expression by adult epithelia, such as pancreatobiliary ducts. More work on the mechanisms underlying small bowel Crohn-related metaplastic changes is certainly needed; however, their apparent relevance for both origin and progression of associated cancers seems worth additional investigation, also considering the putative role played by CK7 in other gastrointestinal diseases, such as Barrett’s oesophagus [47, 48], a known metaplastic carcinogenic lesion, and colorectal cancer [34, 35].

In addition to classic/conventional dysplastic lesions [1–7], several “non-conventional” atypical lesions or growths have been reported to be associated with (and likely precede) cancer development in IBDs of both large and small intestine [12, 19, 30, 49, 50]. We also found such lesions in the mucosa overlying or adjacent to CrD-SBC; indeed, in seven cases, these were in direct continuity with cancer tissue and sometimes with patterns suggestive of direct transition. Thus, despite their polymorphic histology, incompletely defined diagnostic criteria and substantial lack of frank dysplasia, a role for such atypical, non-conventional lesions in the histogenesis of some CrD-SBCs seems likely.

In conclusion, our fairly large retrospective CrD-SBC series showed important heterogeneity of histologic structure and tumour cell phenotype, also paralleled in part by a polymorphism of mucosal precancerous changes. Among the latter, in addition to classic (conventional) dysplasia, CK7-positive and MUC5AC-positive metaplastic lesions, on one hand, and non-conventional, atypical growths, on the other hand, may have a role in Crohn’s disease-promoted cancer histogenesis. In addition, tumour CK7-positive metaplastic phenotype and non-cohesive histotype were both found to predict CrD-SBC patient adverse prognosis. These findings, besides suggesting a distinctive CrD-SBC natural history, are worth considering in future prospective and endoscopic/bioptic studies aiming to gain diagnosis of early tumourigenic lesions, as well as in attempts to provide more appropriate and patient-personalized treatments [22, 42, 51].

## Supplementary information

ESM 1 (PNG 10035 kb)

**Supplementary Figure 1**. A minute intramucosal CrD-SBC (A and enlarged in C, haematoxylin and eosin staining), well demarcated by massive nuclear p53 immunoreactivity (B and D, p53 immunohistochemistry), arises in the background of an extensive non-conventional growth of columnar, eosinophilic cells, which entirely surrounds it, a pattern highly suggestive for a histogenetic link between the two growths. For comparison, note in C), bottom left, a fragment of normal ileal mucosa with goblet cells and in D) the sparse and scattered p53 reactivity of the columnar cells in the non-conventional growth. High resolution image (TIFF 135498 kb)

ESM 2 (PNG 12387 kb)

**Supplementary Figure 2**. A hypermucinous, non-conventional papillary growth (A, haematoxylin and eosin staining), MUC2-reactive (B, MUC2 immunohistochemistry) generates (see a cancer focus in B on the right) and covers a signet-ring type diffuse cancer (C, haematoxylin-eosin) composed of MUC2-positive cells (D, MUC2 immunohistochemistry). High resolution image (TIFF 170428 kb)

ESM 3 (DOCX 9 kb)

## Abbreviations

CI: confidence interval
CrD-SBC: Crohn’s disease-associated small bowel carcinoma
CK7: cytokeratin 7
CK20: cytokeratin 20
HR: hazard ratio
IBD: inflammatory bowel disease
MMR: mismatch repair
MSI: microsatellite instability
no-PID-SBC: small bowel carcinoma arisen sporadically without tumour predisposing immune-inflammatory disease
SBC: small bowel carcinoma
TIL: tumour-infiltrating lymphocytes
UACL: ulcer-associated lineage

## References

[1] Sigel JE, Petras RE, Lashner BA, Fazio VW, Goldblum JR (1999). Intestinal adenocarcinoma in Crohn’s disease: a report of 30 cases with a focus on coexisting dysplasia. Am J Surg Pathol.

[2] Svrcek M, Piton G, Cosnes J, Beaugerie L, Vermeire S, Geboes K, Lemoine A, Cervera P, el-Murr N, Dumont S, Scriva A, Lascols O, Ardizzone S, Fociani P, Savoye G, le Pessot F, Novacek G, Wrba F, Colombel JF, Leteurtre E, Bouhnik Y, Cazals-Hatem D, Cadiot G, Diebold MD, Rahier JF, Delos M, Fléjou JF, Carbonnel F (2014). Small bowel adenocarcinomas complicating Crohn’s disease are associated with dysplasia: a pathological and molecular study. Inflamm Bowel Dis.

[3] Vanoli A, Di Sabatino A, Furlan D (2017). Small bowel carcinomas in coeliac or Crohn’s disease: clinico-pathological, molecular, and prognostic features. A Study From the Small Bowel Cancer Italian Consortium. J Crohns Colitis.

[4] Riddell RH, Goldman H, Ransohoff DF, Appelman HD, Fenoglio CM, Haggitt RC, hren C, Correa P, Hamilton SR, Morson BC, Sommers SC, Yardley JH (1983). Dysplasia in inflammatory bowel disease: standardized classification with provisional clinical applications. Hum Pathol.

[5] Itzkowitz SH, Harpaz N (2004). Diagnosis and management of dysplasia in patients with inflammatory bowel diseases. Gastroenterology.

[6] Simon M, Cosnes J, Gornet JM, GETAID group (2017). Endoscopic detection of small bowel dysplasia and adenocarcinoma in Crohn’s disease: a prospective cohort-study in high-risk patients. J Crohns Colitis.

[7] Grolleau C, Pote NM, Guedj NS, Zappa M, Theou-Anton N, Bouhnik Y, Panis Y, Cazals-Hatem DL (2017). Small bowel adenocarcinoma complicating Crohn’s disease: a single-centre experience emphasizing the importance of screening for dysplasia. Virchows Arch.

[8] Galandiuk S, Rodriguez-Justo M, Jeffery R (2012). Field cancerization in the intestinal epithelium of patients with Crohn’s ileocolitis. Gastroenterology.

[9] Hirsch D, Wangsa D, Zhu YJ, Hu Y, Edelman DC, Meltzer PS, Heselmeyer-Haddad K, Ott C, Kienle P, Galata C, Horisberger K, Ried T, Gaiser T (2018). Dynamics of genome alterations in Crohn’s disease-associated colorectal carcinogenesis. Clin Cancer Res.

[10] Roberts IS, Stoddart RW (1993). Ulcer-associated cell lineage (‘pyloric metaplasia’) in Crohn’s disease: a lectin histochemical study. J Pathol.

[11] Whitcomb E, Liu X, Xiao SY (2014). Crohn enteritis-associated small bowel adenocarcinomas exhibit gastric differentiation. Hum Pathol.

[12] Kilgore SP, Sigel JE, Goldblum JR (2000). Hyperplastic-like mucosal change in Crohn’s disease: an unusual form of dysplasia?. Mod Pathol.

[13] Kaneko Y, Nakamura T, Hayama M, Hosaka N, Akamatsu T, Ota H (2008). Altered expression of CDX-2, PDX-1 and mucin core proteins in “Ulcer-associated cell lineage (UACL)” in Crohn’s disease. J Mol Histol.

[14] Thorsvik S, Bakke I, van Beelen GA (2018). Expression of neutrophil gelatinase-associated lipocalin (NGAL) in the gut in Crohn’s disease. Cell Tissue Res.

[15] Vanoli A, Di Sabatino A, Martino M (2017). Small bowel carcinomas in celiac or Crohn’s disease: distinctive histophenotypic, molecular and histogenetic patterns. Mod Pathol.

[16] Kushima R, Borchard F, Hattori T (1997). A new aspect of gastric metaplasia in Crohn’s disease: bidirectional (foveolar and pyloric) differentiation in so-called ‘pyloric metaplasia’ in the ileum. Pathol Int.

[17] Jun SY, Eom DW, Park H, Bae YK, Jang KT, Yu E, Hong SM (2014). Prognostic significance of CDX2 and mucin expression in small intestinal adenocarcinoma. Mod Pathol.

[18] Stenling R, Lindberg J, Rutegård J (2007). Altered expression of CK7 and CK20 in preneoplastic and neoplastic lesions in ulcerative colitis. APMIS.

[19] Choi WT, Yozu M, Miller GC, Shih AR, Kumarasinghe P, Misdraji J, Harpaz N, Lauwers GY (2020). Nonconventional dysplasia in patients with inflammatory bowel disease and colorectal carcinoma: a multicenter clinicopathologic study. Mod Pathol.

[20] Coit DG, Kelsen D, Tang LH, Erasmus JJ, Gerdes H, Hofstetter WL, Amin MB, Edge SB, Greene FL (2017). Small intestine. AJCC Cancer Staging Manual.

[21] Arpa G, Grillo F, Giuffrida P, Nesi G, Klersy C, Mescoli C, Lenti MV, Lobascio G, Martino M, Latella G, Malvi D, Macciomei MC, Fociani P, Villanacci V, Rizzo A, Ferrero S, Sessa F, Orlandi A, Monteleone G, Biancone L, Cantoro L, Tonelli F, Ciardi A, Poggioli G, Rizzello F, Ardizzone S, Sampietro G, Solina G, Oreggia B, Papi C, D’Incà R, Vecchi M, Caprioli F, Caronna R, D’Errico A, Fiocca R, Rugge M, Corazza GR, Luinetti O, Paulli M, Solcia E, di Sabatino A, Vanoli A (2020). Separation of low- versus high-grade Crohn’s disease-associated small bowel carcinomas is improved by invasive front prognostic marker analysis. J Crohns Colitis.

[22] Vanoli A, Grillo F, Guerini C, Neri G, Arpa G, Klersy C, Nesi G, Giuffrida P, Sampietro G, Ardizzone S, Fociani P, Fiocca R, Latella G, Sessa F, D’Errico A, Malvi D, Mescoli C, Rugge M, Ferrero S, Poggioli G, Rizzello F, Macciomei MC, Santini D, Volta U, de Giorgio R, Caio G, Calabrò A, Ciacci C, D’Armiento M, Rizzo A, Solina G, Martino M, Tonelli F, Villanacci V, Cannizzaro R, Canzonieri V, Florena AM, Biancone L, Monteleone G, Caronna R, Ciardi A, Elli L, Caprioli F, Vecchi M, D’Incà R, Zingone F, D’Odorico A, Lenti MV, Oreggia B, Reggiani Bonetti L, Giannone AG, Orlandi A, Barresi V, Ciccocioppo R, Amodeo G, Biletta E, Luinetti O, Pedrazzoli P, Pietrabissa A, Corazza GR, Solcia E, Paulli M, di Sabatino A (2021). Prognostic role of mismatch repair status, histotype and high-risk pathologic features in stage II small bowel adenocarcinomas. Ann Surg Oncol.

[23] Rizzo F, Vanoli A, Sahnane N, Cerutti R, Trapani D, Rinaldi A, Sellitto A, Ciacci C, Volta U, Villanacci V, Calabrò A, Arpa G, Luinetti O, Paulli M, Solcia E, di Sabatino A, Sessa F, Weisz A, Furlan D (2020). Small-bowel carcinomas associated with celiac disease: transcriptomic profiling shows predominance of microsatellite instability-immune and mesenchymal subtypes. Virchows Arch.

[24] Chiaravalli AM, Feltri M, Bertolini V, Bagnoli E, Furlan D, Cerutti R, Novario R, Capella C (2006). Intratumour T cells, their activation status and survival in gastric carcinomas characterised for microsatellite instability and Epstein-Barr virus infection. Virchows Arch.

[25] Nearchou IP, Kajiwara Y, Mochizuki S, Harrison DJ, Caie PD, Ueno H (2019). Novel internationally verified method reports desmoplastic reaction as the most significant prognostic feature for disease-specific survival in stage II colorectal cancer. Am J Surg Pathol.

[26] Chen ZM, Wang HL (2004). Alteration of cytokeratin 7 and cytokeratin 20 expression profile is uniquely associated with tumorigenesis of primary adenocarcinoma of the small intestine. Am J Surg Pathol.

[27] Kaserer K, Schmaus J, Bethge U, Migschitz B, Fasching S, Walch A, Herbst F, Teleky B, Wrba F (2000). Staining patterns of p53 immunohistochemistry and their biological significance in colorectal cancer. J Pathol.

[28] Ueno H, Murphy J, Jass JR (2000). Tumour ‘budding’ as an index to estimate the potential of aggressiveness in rectal cancer. Histopathology.

[29] Barresi V, Reggiani Bonetti L, Ieni A, Caruso RA, Tuccari G (2017). Poorly differentiated clusters: clinical impact in colorectal cancer. Clin Colorectal Cancer.

[30] Andersen SN, Lovig T, Clausen OP (1999). Villous, hypermucinous mucosa in long standing ulcerative colitis shows high frequency of K-ras mutations. Gut.

[31] Shibahara H, Higashi M, Koriyama C, Yokoyama S, Kitazono I, Kurumiya Y, Narita M, Kuze S, Kyokane T, Mita S, Arai T, Kato T, Yuasa N, Yamaguchi R, Kubota H, Suzuki H, Baba S, Rousseau K, Batra SK, Yonezawa S (2014). Pathobiological implications of mucin (MUC) expression in the outcome of small bowel cancer. PLoS One.

[32] Xue Y, Reid MD, Balci S (2011). Immunohistochemical classification of ampullary carcinomas: critical reappraisal fails to confirm prognostic relevance for recently proposed panels, and highlights MUC5AC as a strong prognosticator. Am J Surg Pathol.

[33] Ushiku T, Arnason T, Fukayama M, Lauwers GY (2014). Extra-ampullary duodenal adenocarcinoma. Am J Surg Pathol.

[34] Harbaum L, Pollheimer MJ, Kornprat P, Lindtner RA, Schlemmer A, Rehak P, Langner C (2011). Keratin 7 expression in colorectal cancer--freak of nature or significant finding?. Histopathology.

[35] Fei F, Li C, Cao Y, Liu K, du J, Gu Y, Wang X, Li Y, Zhang S (2019). CK7 expression associates with the location, differentiation, lymph node metastasis, and the Dukes’ stage of primary colorectal cancers. J Cancer.

[36] Wu Y, Grabsch H, Ivanova T, Tan IB, Murray J, Ooi CH, Wright AI, West NP, Hutchins GGA, Wu J, Lee M, Lee J, Koo JH, Yeoh KG, van Grieken N, Ylstra B, Rha SY, Ajani JA, Cheong JH, Noh SH, Lim KH, Boussioutas A, Lee JS, Tan P (2013). Comprehensive genomic meta-analysis identifies intra-tumoural stroma as a predictor of survival in patients with gastric cancer. Gut.

[37] Wang LM, Silva MA, D'Costa Z (2016). The prognostic role of desmoplastic stroma in pancreatic ductal adenocarcinoma. Oncotarget.

[38] Kim A, Bae YK, Gu MJ (2013). Epithelial-mesenchymal transition phenotype is associated with patient survival in small intestinal adenocarcinoma. Pathology.

[39] Isella C, Terrasi A, Bellomo SE, Petti C, Galatola G, Muratore A, Mellano A, Senetta R, Cassenti A, Sonetto C, Inghirami G, Trusolino L, Fekete Z, de Ridder M, Cassoni P, Storme G, Bertotti A, Medico E (2015). Stromal contribution to the colorectal cancer transcriptome. Nat Genet.

[40] Guinney J, Dienstmann R, Wang X, de Reyniès A, Schlicker A, Soneson C, Marisa L, Roepman P, Nyamundanda G, Angelino P, Bot BM, Morris JS, Simon IM, Gerster S, Fessler E, de Sousa E Melo F, Missiaglia E, Ramay H, Barras D, Homicsko K, Maru D, Manyam GC, Broom B, Boige V, Perez-Villamil B, Laderas T, Salazar R, Gray JW, Hanahan D, Tabernero J, Bernards R, Friend SH, Laurent-Puig P, Medema JP, Sadanandam A, Wessels L, Delorenzi M, Kopetz S, Vermeulen L, Tejpar S (2015). The consensus molecular subtypes of colorectal cancer. Nat Med.

[41] Solcia E, Klersy C, Vanoli A, Grillo F, Manca R, Tava F, Luinetti O, Fiocca R (2013). The contribution of cell phenotype to the behavior of gastric cancer. Gastric Cancer.

[42] Giuffrida P, Arpa G, Grillo F, Klersy C, Sampietro G, Ardizzone S, Fociani P, Fiocca R, Latella G, Sessa F, D’Errico A, Malvi D, Mescoli C, Rugge M, Nesi G, Ferrero S, Furlan D, Poggioli G, Rizzello F, Macciomei MC, Santini D, Volta U, de Giorgio R, Caio G, Calabrò A, Ciacci C, D’Armiento M, Rizzo A, Solina G, Martino M, Tonelli F, Villanacci V, Cannizzaro R, Canzonieri V, Florena AM, Biancone L, Monteleone G, Caronna R, Ciardi A, Elli L, Caprioli F, Vecchi M, D’Incà R, Zingone F, D’Odorico A, Lenti MV, Oreggia B, Reggiani Bonetti L, Astegiano M, Biletta E, Cantoro L, Giannone AG, Orlandi A, Papi C, Perfetti V, Quaquarini E, Sandri G, Silano M, Usai P, Barresi V, Ciccocioppo R, Luinetti O, Pedrazzoli P, Pietrabissa A, Viglio A, Paulli M, Corazza GR, Solcia E, Vanoli A, di Sabatino A (2020). PD-L1 in small bowel adenocarcinoma is associated with etiology and tumor-infiltrating lymphocytes, in addition to microsatellite instability. Mod Pathol.

[43] Simmini S, Bialecka M, Huch M, Kester L, van de Wetering M, Sato T, Beck F, van Oudenaarden A, Clevers H, Deschamps J (2014). Transformation of intestinal stem cells into gastric stem cells on loss of transcription factor Cdx2. Nat Commun.

[44] Kirchner T, Müller S, Hattori T, Mukaisyo K, Papadopoulos T, Brabletz T, Jung A (2001). Metaplasia, intraepithelial neoplasia and early cancer of the stomach are related to dedifferentiated epithelial cells defined by cytokeratin-7 expression in gastritis. Virchows Arch.

[45] Kojima J, Fukuda A, Taira H, Kawasaki T, Ito H, Kuji N, Isaka K, Umezawa A, Akutsu H (2017). Efficient production of trophoblast lineage cells from human induced pluripotent stem cells. Lab Investig.

[46] Tajima Y, Ito K, Umino A, Wilkinson AC, Nakauchi H, Yamazaki S (2017). Continuous cell supply from Krt7-expressing hematopoietic stem cells during native hematopoiesis revealed by targeted in vivo gene transfer method. Sci Rep.

[47] Ormsby AH, Goldblum JR, Rice TW, Richter JE, Falk GW, Vaezi MF, Gramlich TL (1999). Cytokeratin subsets can reliably distinguish Barrett’s esophagus from intestinal metaplasia of the stomach. Hum Pathol.

[48] Jiang M, Li H, Zhang Y, Yang Y, Lu R, Liu K, Lin S, Lan X, Wang H, Wu H, Zhu J, Zhou Z, Xu J, Lee DK, Zhang L, Lee YC, Yuan J, Abrams JA, Wang TC, Sepulveda AR, Wu Q, Chen H, Sun X, She J, Chen X, Que J (2017). Transitional basal cells at the squamous-columnar junction generate Barrett’s oesophagus. Nature.

[49] Gui X, Köbel M, Ferraz JG (2020). Histological and molecular diversity and heterogeneity of precancerous lesions associated with inflammatory bowel diseases. J Clin Pathol.

[50] Gui X, Iacucci M, Ghosh S, Ferraz JGP, Lee S (2020). Revisiting the distinct histomorphologic features of inflammatory bowel disease-associated neoplastic precursor lesions in the SCENIC and post-DALM Era. Hum Pathol.

[51] Liao X, Li G, McBride R, Houldsworth J, Harpaz N, Polydorides AD (2020). Clinicopathological and molecular characterisation of Crohn’s disease-associated small bowel adenocarcinomas. J Crohns Colitis.

