# Supplementary figures and images for: Prognostic relevance and putative histogenetic role of cytokeratin 7 and MUC5AC expression in Crohn’s disease-associated small bowel carcinoma
Source: Virchows Arch. 2021 May 8;479(4):667–78. doi: 10.1007/s00428-021-03109-2 (PMC8516779; doi:10.1007/s00428-021-03109-2)

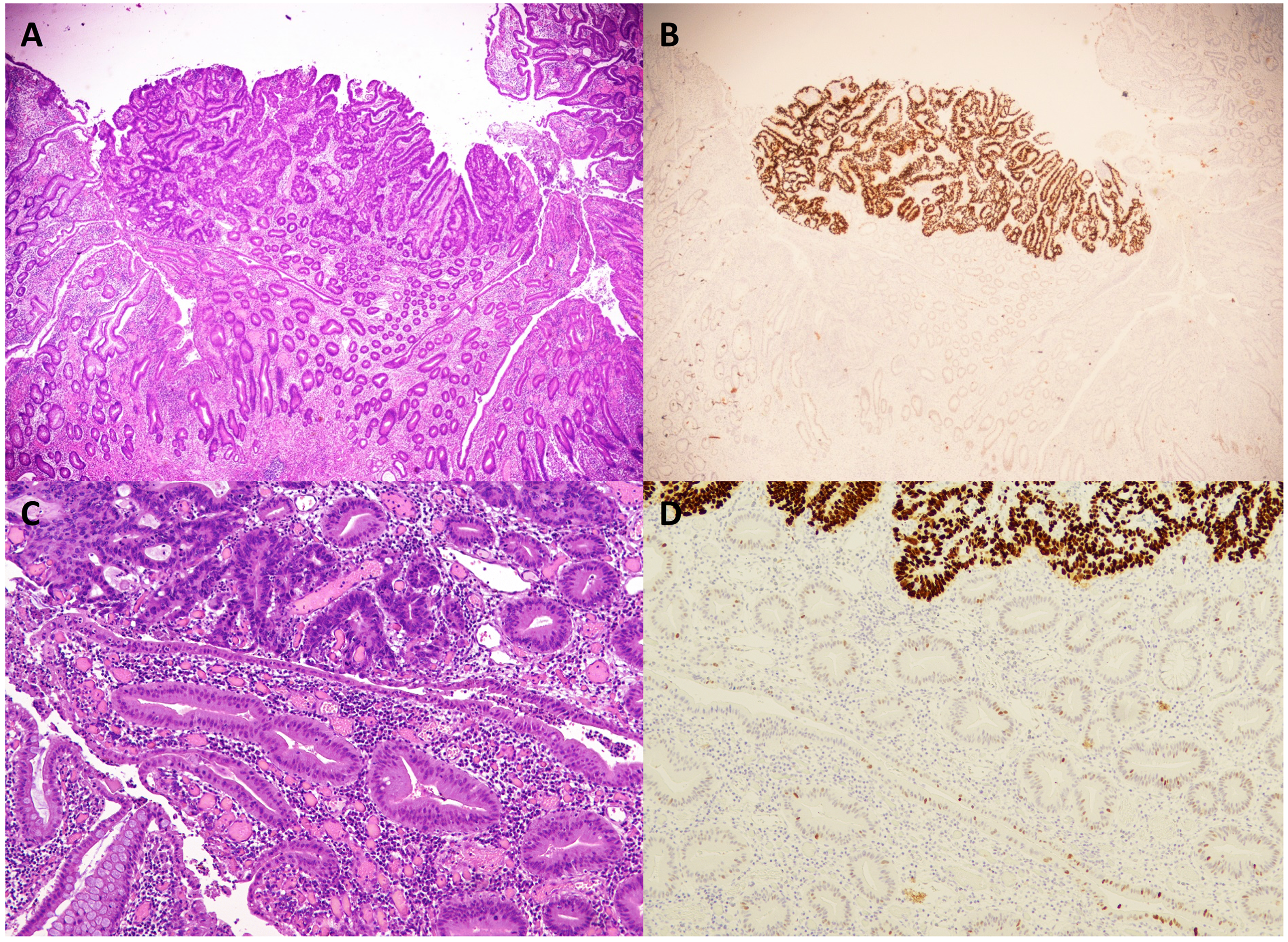

Supplement: Supplementary file 1 — (PNG 10035 kb) [file 428_2021_3109_Fig4_ESM.png]

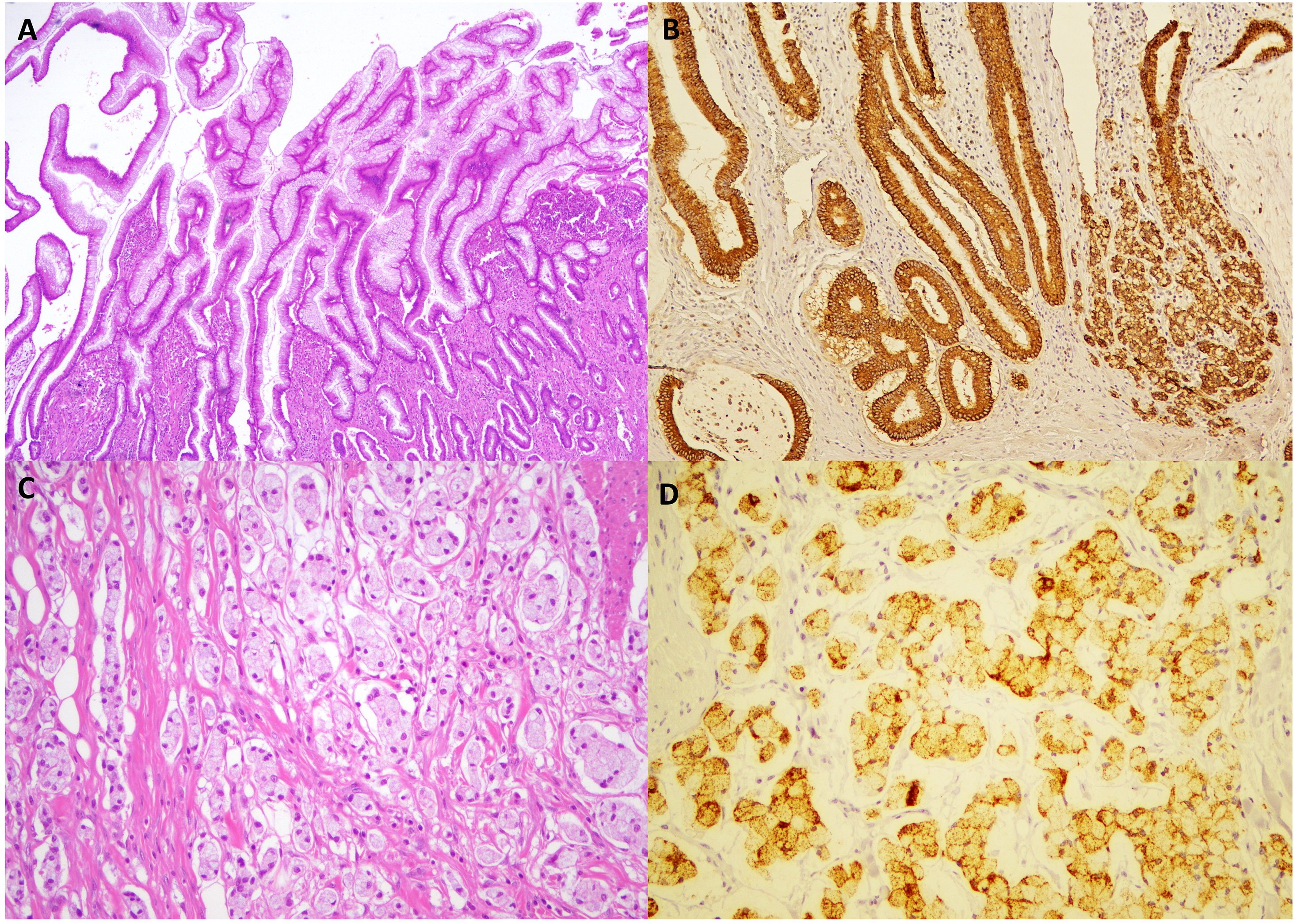

Supplement: Supplementary file 3 — (PNG 12387 kb) [file 428_2021_3109_Fig5_ESM.png]
