# Supplementary material for: Prognostic relevance and putative histogenetic role of cytokeratin 7 and MUC5AC expression in Crohn’s disease-associated small bowel carcinoma
Source: Virchows Arch. 2021 May 8;479(4):667–78. doi: 10.1007/s00428-021-03109-2 (PMC8516779; doi:10.1007/s00428-021-03109-2)
Supplement: Supplementary file 5 — (DOCX 9 kb) [file 428_2021_3109_MOESM3_ESM.docx]

**Supplementary Table 1.** Analysis of histotype and MUC2, cytokeratin 20 and p53 expression of Crohn’s disease-associated small bowel carcinomas.

| **Histotype** | **N (%)** | **CK20+** | **MUC2+** | **P53+** |
| --- | --- | --- | --- | --- |
| **Cohesive, N (%)** | 29/52 (56) | 14/29 (48) | 10/18 (55) | 17/29 (59) |
| **Non-cohesive, N (%)**  Diffuse, N (%)  Mixed, N (%) | 23/52 (44)  11/52 (21)  12/52 (23) | 14/23 (61)  7/11 (64)  7/12 (58) | 8/15 (53)  3/6 (50)  5/9 (55) | 10/23 (43)  3/11 (27)  7/12 (58) |
| **Total, N (%)** | 52/52 (100) | 28/52 (54)* | 18/33 (54)** | 27/52 (52)° |

CK20 and MUC2 were scored as positive if ≥10% of tumor cells were stained; p53 was scored as positive (widespread) if >50% of tumour cells were stained. Only 33 CrD-SBCs were tested for MUC2, due to depletion of sufficient representative tumour sections. * Significant difference in CK20 expression versus no-PID-SBCs (p=0.012). ** No significant difference in MUC2 expression versus 28 no-PID-SBCs (p=0.611). ° No significant difference in p53 expression versus no-PID-SBCs (p=0.371).
